# Relationship between maximum voided volume obtained by bladder diary compared to contemporaneous uroflowmetry in men and women

**DOI:** 10.1590/S1677-5538.IBJU.2021.0211

**Published:** 2021-06-25

**Authors:** Kevin Rychik, Lucas Policastro, Jeffrey Weiss, Jerry Blaivas

**Affiliations:** 1 Sackler School of Medicine New YorkNY USA Sackler School of Medicine, New York, NY, USA; 2 Institute for Bladder and Prostate Research New YorkNY USA Institute for Bladder and Prostate Research, New York, NY, USA; 3 Wayne Memorial Hospital JesupGA USA Wayne Memorial Hospital, Jesup, GA, USA; 4 New York Downstate Health Sciences University State University New YorkNY USA State University of New York Downstate Health Sciences University, New York, NY, USA; 5 Icahn School of Medicine at Mount Sinai New YorkNY USA Icahn School of Medicine at Mount Sinai, New York, NY, USA

**Keywords:** Urinary Bladder, Lower Urinary Tract Symptoms, Retrospective Studies

## Abstract

**Introduction:**

The 24-hour bladder diary is considered to be the gold standard for evaluating maximum voided volume (MVV). However, we observed that patients often have a greater MVV during office uroflowmetry than that seen in the bladder diary. The purpose of this study is to compare these two non-invasive methods by which MVV can be determined - at the time of uroflowmetry (Q-MVV), or by 24hour bladder diary (BD-MVV).

**Materials and Methods:**

This was an Institutional Review Board approved retrospective study of patients evaluated for LUTS who completed a 24hour bladder diary and contemporaneous uroflowmetry. For Q-MVV, the patient was instructed to wait to void until their bladder felt full. Sample means were compared, and Pearson's correlations were calculated between the Q-MVV and BD-MVV data across the total sample, women, and men.

**Results:**

Seven hundred seventy one patients with LUTS completed bladder diaries. Of these, 400 patients, 205 women and 195 men, had contemporaneous Q-MVV. Mean BD-MVV was greater than mean Q-MVV. However, Q-MVV was larger in a sizable minority of patients. There was a weak correlation between BD-MVV and Q-MVV. Furthermore, there was a difference ≥50% between Q-MVV and BD-MVV in 165 patients (41%).

**Conclusions:**

The data suggest that there is a difference between the two measurement tools, and that the BD-MVV was greater than Q-MVV. For a more reliable assessment of MVV, this study suggests that both Q-MVV and BD-MVV should be assessed and that the larger of the two values is a more reliable assessment of MVV.

## INTRODUCTION

Lower urinary tract symptoms (LUTS) are subjective indicators of lower urinary tract dysfunction. Clinical guidelines for evaluation of LUTS in men and women require a focused history and physical examination. Both bladder diaries and uroflow are adjunctive tools that may be considered as part of the diagnostic evaluation (1, 2).

Most guidelines recommend that bladder diaries be kept for one to seven days with the caveat that the longer the diary, the more reliable the data, but the poorer the patient compliance (3, 4). For the diary, the patient is instructed to record the time and amount of each void for at least twenty-four hours, and contemporaneous symptoms with other annotations are recorded for each void. In some cases, oral intake may also be recorded in the bladder diary (5, 6). One of the various metrics that can be determined from the voiding diary is the maximum voided volume (BD-MVV). This is an important determinant of voiding behavior and can be used as a diagnostic tool, benchmark for behavior modification, and/or a metric of treatment success (7-10). However, not all patients are willing or able to perform a voiding diary. An alternative method to estimate MVV is during office uroflowmetry (6) when the patient is instructed to wait until the bladder feels full - the MVV obtained at the time of uroflow (Q-MVV) (11).

The purpose of this study is to compare Q-MVV to BD-MVV, and to assess the differences between them in patients with reported LUTS.

## MATERIALS AND METHODS

This was an institutional review board approved retrospective study of men and women evaluated for LUTS. A database of 771 patients evaluated for LUTS who completed a 24-hour bladder diary independently using a smartphone application (weShare® URO from Symptelligence.com) was searched for inclusion into the study. Exclusion criteria were incomplete/erroneous diary entries or bladder diaries without a contemporaneous uroflowmetry. Uroflowmetry was performed routinely for both men and women with measurement of flow and voided volume. BD-MVV is the volume of largest void obtained during a 24-hour assessment period. Q-MVV is the voided volume in the clinical setting.

The following data were extracted from the bladder diary and uroflowmetry for each patient: BD-MVV, maximum flow rate (Qmax), and Q-MVV. BD-MVV is the volume of largest void obtained during a 24-hour assessment period recorded independently by the patient in a 24-hour bladder diary via the smartphone application. Qmax is the maximum flow rate measured by uroflowmetry in the clinical setting (12). Q-MVV is the maximum voided volume measured by uroflowmetry in the clinical setting.

The uroflowmetry data were considered contemporaneous if they were recorded within 3 months of the BD-MVV provided that there were no new treatments or change in symptoms. The contemporaneous Q-MVV was collected in the clinical setting per each patient after they were instructed to wait to void until the bladder felt full. A measure of Q-MVV with a full bladder was designed to simulate a natural void to be compared contemporaneously to the BD-MVV. When multiple uroflowmetries were available, the Q-MVV with the highest Qmax was used. When multiple bladder diaries were completed, the earliest one was used. Sample means were compared via independent two sample t-tests, standard deviation, maximum and minimum values, and Pearson's correlations were calculated between the Q-MVV and BD-MVV data across the total sample, women, and men.

## RESULTS

Seven hundred seventy one patients with LUTS, ages 20-94 years, completed bladder diaries. Of these, 400 patients, 205 women and 195 men, had contemporaneous uroflowmetry data inputted to date. Table-1 shows a comparison of BD-MVV and Q-MVV data in the total group, women, and men. The mean BD-MVV was greater than the Q-MVV in the total group. The BD-MVV was larger than the Q-MVV in 317 patients total (79%), and the Q-MVV was larger in 83 of the patients (21%).

**Table 1:** Results of independent two sample t-tests comparing mean BD-MVV to Q-MVV in the total sample, and across women, and men.

	N	Mean (mL)	SD (mL)	Δ (mL)	Min. (mL)	Max. (mL)	t	p
**BD-MVV Total**	400	340.46	147.83		50	900		
**Q-MVV Total**	400	216.58	152.11	+123.88	23	1000	1.96	<0.001
**BD-MVV Women**	205	321.67	151.42		50	900		
**Q-MVV Women**	205	218.16	149.61	+103.51	23	813	1.97	<0.001
**BD-MVV Men**	195	357.77	142.37		84	900		
**Q-MVV Men**	195	214.92	155.07	+142.85	23	1000	1.97	<0.001

**Figure 1:**
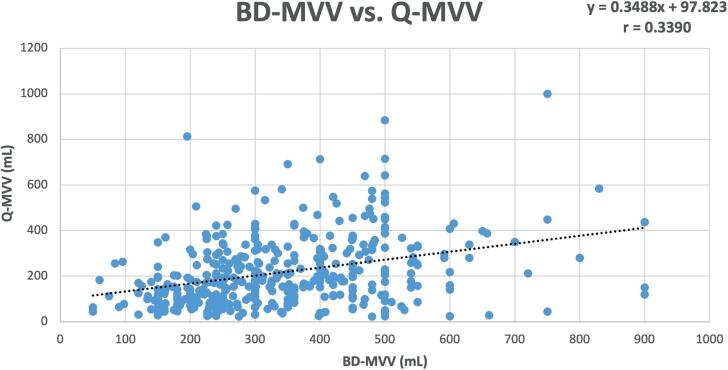
Scatterplot of BD-MVV vs. Q-MVV (n=400).

A scatter plot depicts the relationship between BD-MVV and Q-MVV shown in Figure-1. Analysis of the relationship was performed using a Pearson's correlation. The Pearson's r=34, indicating a weak positive correlation.

**Figure 2:**
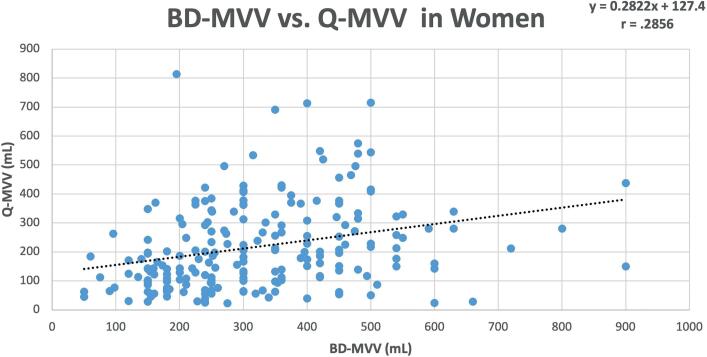
Scatterplot of bladder diary BD-MVV vs. Q-MVV in women (n=205).

**Figure 3:**
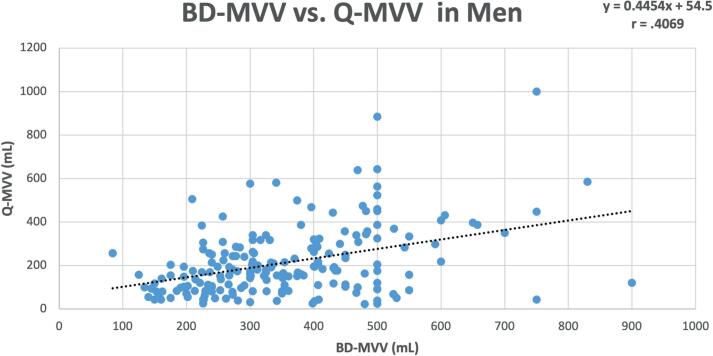
Scatterplot of bladder diary BD-MVV vs. Q-MVV in men (n=195).

Data for women and men is depicted in Figures 2 and 3 respectively.

The difference between BD-MVV and Q-MVV as a percentage of the larger of the two measurements was calculated for each of the 400 patients. In 165 patients, or 41% of the total sample, there was a difference in MVV ≥50% between Q-MVV and BD-MVV, and in 260 patients, (65%), there was a difference ≥25%.

## DISCUSSION

Tissot (2008) published mean values for 24-hour voiding frequency, 24-hour voided volume, maximum and minimum voided volumes and volumes per void for 92 (aged 21-84 years) men and 161 women (aged 21-84 years) without LUTS. The mean BD-MVV for men and women, were 500mL and 514mL respectively (13, 14). In our sample of men with LUTS, the BD-MVV and Q-MVV were 357.77mL and 214.92mL respectively. In our sample of women with LUTS, the BD-MVV and Q-MVV were 321.67mL and 218.16mL respectively. This is consistent with the notion that maximum voided volume is reduced in patients with LUTS.

It is well documented that, to a large degree, uroflow is dependent on bladder volume - the larger bladder volume, the greater the flow (15, 16). For this reason, patients are usually instructed to wait until the bladder is full before obtaining a uroflow.

Although the mean BD-MVV was larger than Q-MVV, the Q-MVV was larger in 21% of patients. Moreover, when calculating the difference between the two measurement tools as a percentage of the larger value, we found a discrepancy of more than 50% in 165 of the 400 patients in the sample. This suggests an estimation of MVV may be inaccurate by 50% or more if only one measurement tool is used. Furthermore, there was only a weak positive correlation between the two tools. These findings are significant because an assessment of patient's MVV through a singular use of BD-MVV or Q-MVV may be lacking.

An accurate estimation of maximum voided volume is important for a number of reasons. Firstly, in theory, if MVV is increased, for any given condition, the number of voids per 24 hours could be decreased provided that the 24hour voided volume does not change significantly. Secondly, changes in MVV provide an outcome metric by which the success or failure of treatment is judged (10). In a phase two study of combination therapy for patients with overactive bladder, the primary efficacy outcome measure was an increase in mean volume voided per micturition (10). Thirdly, the relation between symptom severity, MVV, and bladder capacity provides a metric for understanding the underlying pathophysiology for developing phenotypes (17). Finally, MVV provides information that is useful for developing diagnostic and treatment pathways in future research. For example, in a recent study, patients with a low MVV (<150mL), who voided less than 1L in 24 hours, were older and more likely to have indicators of urethral obstruction or detrusor underactivity than those with an MVV >150mL and polyuria (18).

Uroflowmetry has long been considered the first line screening test for most patients with suspected urethral obstruction (19). In contrast to uroflowmetry, the bladder diary is likely to be more representative of the natural home setting; and, as expected, this study confirmed a discrepancy between information obtained through both methods. Advantages of uroflowmetry include a controlled administration environment, less interpretation time, and it is less prone to human error. Disadvantages include cost, and the fact that many patients with LUTS, for matters of expediency, do not wait until the bladder is full before voiding for uroflowmetry. This was born out in the current study insofar as the MVV obtained by bladder diary was greater than that obtained at the time of uroflowmetry. For both the bladder diary and uroflowmetry, patient compliance is needed for filling out the diary effectively or arriving to the clinic with a full bladder.

The primary weakness of this study is that it was retrospective. Uroflowmetry was performed as a routine procedure - not as a specifically targeted measurement of bladder capacity. As such, patients may not have been counseled in a consistent fashion about the meaning of comfortably full. The error incurred by could be an underestimate of the frequency with which the Q-MVV exceeds the BD-MVV. A second weakness is that the de-identified database did not allow correlation with LUTS questionnaires, clinical diagnoses or medication.

## CONCLUSION

The data suggest that there is a difference between the two measurement tools, and that the maximum voided volume recorded in a bladder diary (BD-MVV) was greater than that obtained at the time of uroflow. (Q-MVV). For a more reliable assessment of MVV, this study suggests that both Q-MVV and BD-MVV should be assessed and that the larger of the two values is a more reliable assessment.
